# C-X-C Motif Chemokine 3 Promotes the Inflammatory Response of Microglia after *Escherichia coli*-Induced Meningitis

**DOI:** 10.3390/ijms241310432

**Published:** 2023-06-21

**Authors:** Xinyi Qu, Beibei Dou, Ruicheng Yang, Chen Tan, Huanchun Chen, Xiangru Wang

**Affiliations:** 1 National Key Laboratory of Agricultural Microbiology, College of Veterinary Medicine, Huazhong Agricultural University, Wuhan 430070, China; quxinyi@webmail.hzau.edu.cn (X.Q.); doubeibei714@163.com (B.D.); yangruicheng@mail.hzau.edu.cn (R.Y.); tanchen@mail.hzau.edu.cn (C.T.); chenhch@mail.hzau.edu.cn (H.C.); 2 Key Laboratory of Preventive Veterinary Medicine in Hubei Province, The Cooperative Innovation Center for Sustainable Pig Production, Wuhan 430070, China; 3 Frontiers Science Center for Animal Breeding and Sustainable Production, Wuhan 430070, China; 4 Key Laboratory of Development of Veterinary Diagnostic Products, Ministry of Agriculture of the People’s Republic of China, Wuhan 430070, China; 5 International Research Center for Animal Disease, Ministry of Science and Technology of the People’s Republic of China, Wuhan 430070, China

**Keywords:** *Escherichia coli*, meningitis, inflammation, central nervous system, chemokines

## Abstract

Meningitis is a major clinical manifestation of *Escherichia coli* (*E. coli*) infection characterized by inflammation of the meninges and subarachnoid space. Many chemokines are secreted during meningitic *E. coli* infection, of which C-X-C motif chemokine 3 (CXCL3) is the most highly expressed. However, it is unclear how CXCL3 plays a role in meningitic *E. coli* infection. Therefore, this study used in vitro and in vivo assays to clarify these contributions and to identify novel therapeutic targets for central nervous system inflammation. We found a significantly upregulated expression of CXCL3 in human brain microvascular endothelial cells and U251 cells after meningitic *E. coli* infection, and the CXCL3 receptor, C-X-C motif chemokine receptor 2 (CXCR2), was expressed in microglia. Furthermore, CXCL3 induced *M1* microglia by selectively activating mitogen-activated protein kinases signaling and significantly upregulating tumor necrosis factor-α (TNF-α), interleukin (IL)-1β, IL-6, nitric oxide synthase 2 (NOS2), and cluster of differentiation 86 (CD86) expression levels, promoting an inflammatory response. Our findings clarify the role of CXCL3 in meningitic *E. coli*-induced neuroinflammation and demonstrate that CXCL3 may be a potential therapeutic target for future investigation and prevention of *E. coli*-induced neuroinflammation.

## 1. Introduction

A common infectious disease that affects the central nervous system (CNS) is bacterial meningitis. Organisms that cause meningitis include *Escherichia coli*, *Haemophilus influenzae*, *Streptococcus pneumoniae*, and *Neisseria meningitidis*. It is possible for *E. coli* to infect the CNS and cause neuroinflammation [1]. The blood-brain barrier (BBB) is a structural and functional barrier formed by endothelial cells interacting with pericytes, astrocytes, neurons, and microglia [2,3]. Various molecules pass through it to and from the brain, maintaining the neural microenvironment and protecting the brain from microbes and toxins in the blood [4]. The brain microvascular endothelial cells (BMECs) regulate molecular transport between the bloodstream and the brain in order to maintain a highly controlled neurovascular environment for the proper functioning of neuronal circuits [5]. Astrocytes play an important role in neurodegenerative diseases by contributing to the dynamic regulation of the neural system [6].

Microglia play a critical role in supporting key functions in the CNS [7]. The majority of CNS diseases involve microglia, which convert from a resting/surveillance state in the normal brain to a fully active state in the diseased brain [8]. When the brain parenchyma is infected or stimulated, microglia become active and are involved in a variety of CNS disorders, such as brain trauma, stroke, and Parkinson’s disease; thus, they have contributed significantly to neurological diseases. Studies have shown that, similar to macrophages, microglia have two distinct activation phenotypes: classical (*M1*) and alternative (*M2*). *M1* microglia are more likely to induce neuronal death than *M2* microglia. Collectively, these two microglia activation phenotypes, neurotoxicity, and neuroprotection after activation play important roles in disease pathogenesis.

A chemokine is a chemotactic cytokine that influences the migration patterns and positioning of immune cells [9]. Chemokines play an important role in pathophysiological processes, such as in inflammation, angiogenesis, and asthma [10]. Many cytokines are secreted during meningitic *E. coli* infection, including the chemokine C-X-C motif chemokine 3 (CXCL3), also called growth-related oncogene γ (GRO-γ) or macrophage inflammatory protein 2 (MIP-2) [11,12]. CXCL3 is a neutrophil-activating chemokine that belongs to the growth-related oncogene subfamily of CXC chemokines, which exert their biological roles through the chemokine receptors, C-X-C motif chemokine receptors (CXCRs) 1 and 2 [13,14]. In 2000, Addison et al. demonstrated that CXCR2 mediates the biological functions of CXCL3 [15].

Macrophages, osteoblasts, airway epithelial cells, and dendritic cells secrete CXCL3. In addition, CXCL3 promotes blood vessel formation, tumor cell growth, cancer cell migration, cluster of differentiation (CD) 31 vascular cell infiltration, and smooth muscle cell migration [16]. It is still unclear how CXCL3 contributes to meningitis. Therefore, this study investigated the function of CXCL3 in meningitic *E. coli* infection to identify novel therapeutic targets for CNS inflammation.

## 2. Results

### 2.1. Meningitic E. coli Significantly Stimulates CXCL3 Expression In Vivo and In Vitro

**Figure 1:**
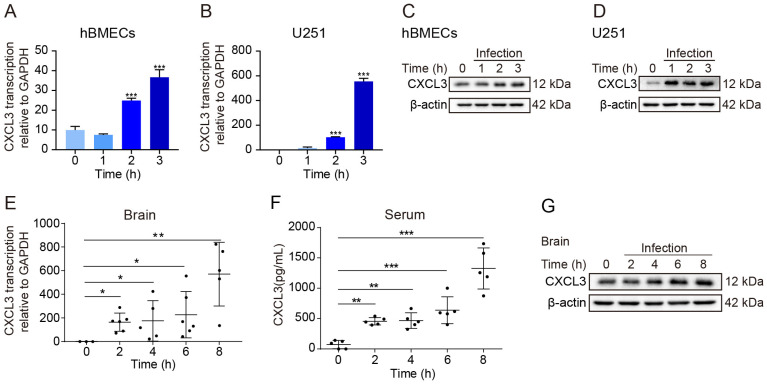
Meningitic *E. coli* infection promotes C-X-C motif chemokine 3 (CXCL3) expression in vivo and in vitro. (**A**,**C**) CXCL3 transcription levels (n = 3) in (**A**) human brain microvascular endothelial cells (hBMECs) and (**B**) U251 cells after meningitic *E. coli* infection at a multiplicity of infection (MOI) of 10 for 1, 2, and 3 h real-time polymerase chain reaction (PCR). (**C**,**D**) CXCL3 immunoblot results from whole cell extracts after infecting (**C**) hBMECs or (**D**) U251 cells after meningitic *E. coli* infection at an MOI of 10 for 1, 2, and 3 h. (**E**) CXCL3 transcription levels (brain lysates; real-time PCR) and (**F**) serum CXCL3 concentrations after meningitic *E. coli* infection (intravenous injection) (n = 5) at 1 × 10^7^ colony forming units for 2, 4, 6, and 8 h. (**G**) CXCL3 protein expression from challenged mice (brain lysates; western blot). Data were presented as mean ± SD from three independent assays. *p <* 0.05 (*) was considered statistically significant; *p* < 0.01 (**) and *p* < 0.001 (***) indicated extremely significant differences.

Previous RNA sequencing data demonstrated upregulated CXCL3 expression in hBMECs and U251 cells [17,18]. As a result of bacterial challenge, hBMEC transcription of CXCL3 increased significantly over time and was sustained (Figure 1A). Furthermore, meningitic *E. coli* infection promoted CXCL3 protein expression (Figure 1B). CXCL3 expression was also upregulated in U251 cells after the bacterial challenge (Figure 1C,D). CXCL3 transcription increased rapidly 1 h post infection, especially in the U251 cells; transcription in these cells, was considerably higher than that in hBMECs.

The brains of mice with meningitic *E. coli* infection also showed significant and time-dependent increases in CXCL3 transcription (Figure 1E), as did the serum CXCL3 protein levels (Figure 1F). Furthermore, serum CXCL3 expression increased 2 h post infection and continued increasing time-dependently (Figure 1G). In summary, treatment with meningitic *E. coli* promoted CXCL3 expression in vitro and in vivo.

### 2.2. Microglia Express CXCR2 during Meningitic E. coli Infection

**Figure 2:**
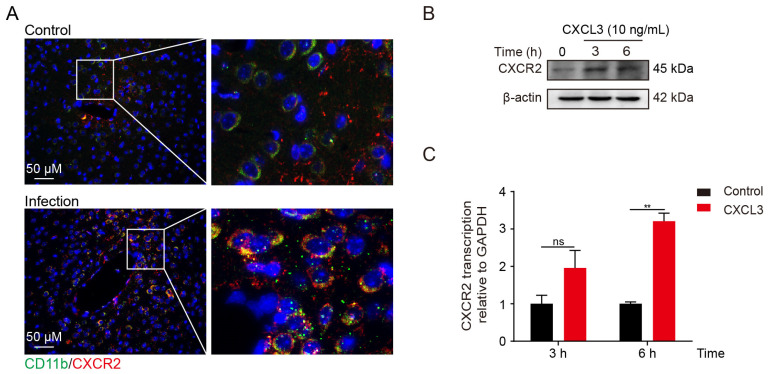
C-X-C motif chemokine receptor 2 (CXCR2) is expressed in microglia. (**A**) Immunofluorescence results of CXCR2 expression (red) and CD11b (green, microglia) from the brain of mice after meningitic *E. coli* infection. Scale bar, 50 µm. (**B**) CXCR2 protein expression in BV2 cells in response to 3 or 6 h of C-X-C motif chemokine 3 (CXCL3) treatment (10 ng/mL) (western blot). (**C**) CXCR2 transcription levels in BV2 cells in response to 3 or 6 h of CXCL3 treatment (10 ng/mL) (n = 3; real-time polymerase chain reaction). GAPDH was used as an internal control for normalization. Data were presented as mean ± SD from three independent assays. *p* < 0.01 (**) indicated extremely significant differences; ns, not significant.

High CXCL3 levels have been detected in mouse brain tissue after infection, and CXCL3 interacts with CXCR2 (the receptor) to produce a range of biological effects. Therefore, we examined the expression of CXCR2 in the mouse brain by immunofluorescence. Complement component receptor-3 alpha (CD11b), a classical microglia marker, was used to detect the location of CXCR2 in the brain; it was expressed in microglia (Figure 2A). Moreover, we observed elevated CXCR2 expression levels in BV2 cells after 3 h of stimulation with recombinant mouse CXCL3 (Figure 2B). Consistently, CXCR2 messenger RNA transcription and protein levels were time-dependently upregulated in response to CXCL3 (Figure 2C). Therefore, we propose that meningitic *E. coli* infection facilitates CXCL3 expression and acts on microglia.

### 2.3. CXCL3 Facilitated Microglia Polarization towards a Pro-Inflammatory Profile

**Figure 3:**
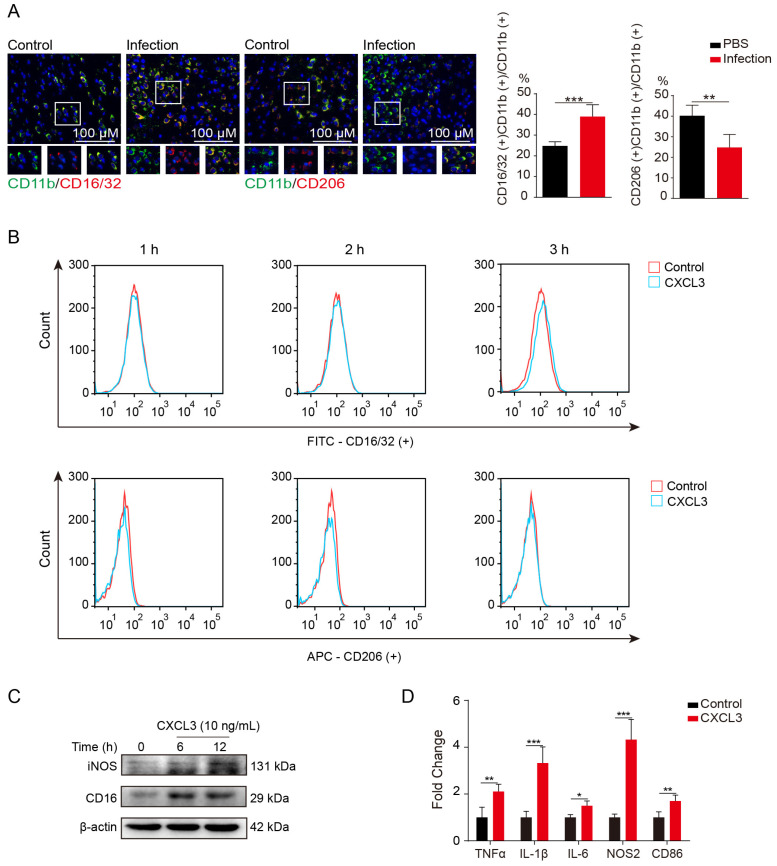
C-X-C motif chemokine 3 (CXCL3) facilitates microglia polarization towards an inflammatory profile. (**A**, **left**) Cluster of differentiation (CD11b (green), CD16/32 (red), and CD206 (red) immunofluorescence results of mice brain with and without meningitic *E. coli* infection for 6 h. Scale bar, 100 µm. (**A**, **right**) Quantitative analysis of CD11b and CD16/32 double-positive and CD11b and CD206 double-positive cells. PBS, phosphate-buffered saline (control). (**B**) CD16/32 and CD206 protein expression levels measured by flow cytometry after 1, 2, or 3 h of pre-treatment with CXCL3 (10 ng/mL). (**C**) Protein expression levels of pro-inflammatory markers (inducible nitric oxide synthase [iNOS], CD16/32) in BV2 cells in response to CXCL3 (10 ng/mL) stimulation (western blot). (**D**) RNA expression profiles of pro-inflammatory markers (tumor necrosis factor-alpha [TNF-α], interleukin [IL]-1β, IL-6, nitric oxide synthase 2 [NOS2], and cluster of differentiation 86 [CD86]) in BV2 cells in response to CXCL3 (10 ng/mL) stimulation for 6 h (n = 3; quantitative real-time polymerase chain reaction). GAPDH was used as an internal control for normalization. Data were presented as mean ± SD from three independent assays. *p* < 0.05 (*) was considered statistically significant; *p* < 0.01 (**) and *p* < 0.001 (***) indicated extremely significant differences.

Microglia become active after stimulation. We therefore investigated whether CXCL3 affects the polarization of microglia toward a pro-inflammatory phenotype in meningitic *E. coli* infection. Compared to control mice, meningitic *E. coli* infection mice had a significant increase in CD16/32 and CD11b double-positive microglia, but a significant decrease in CD206 and CD11b double-positive microglia (Figure 3A). To determine the effect of CXCL3 on the polarization of BV2 cells, we used flow cytometry to detect CD16/32 and CD206 expression and found that CXCL3 (10 ng/mL) treatment for 3 h significantly increased the percentage of CD16/32 positive microglia compared to that in the PBS group, but the percentages of CD206 positive microglia were consistent between the treatment and PBS groups (Figure 3B). These results indicate that CXCL3 induces *M1* microglia polarization.

We also investigated whether CXCL3 induces BV2 cells to express pro-inflammatory factors. We found that pro-inflammatory markers (inducible nitric oxide synthase [iNOS] and CD16) were upregulated by CXCL3 stimulation (Figure 3C; western blot). Additionally, we found significant upregulation of tumor necrosis factor (TNF)-α, interleukin (IL)-1β, IL-6, iNOS, and cluster of differentiation 86 (CD86) (the mannose receptor) expression in BV2 cells stimulated with recombinant mouse CXCL3 for 6 h (Figure 3D). These findings suggest that CXCL3 facilitates the inflammatory response of microglia during meningitic *E. coli* infection.

### 2.4. CXCL3 Regulates Microglia Polarization via the Extracellular Signal-Regulated Protein Kinases 1 and 2 (ERK1/2) Pathway

**Figure 4:**
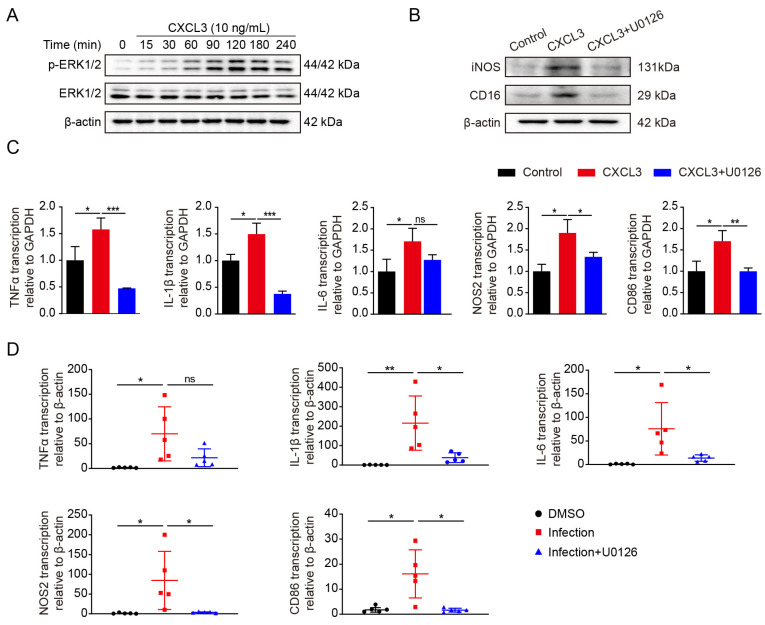
C-X-C motif chemokine 3 (CXCL3) activates the extracellular signal-regulated protein kinases 1 and 2 (ERK1/2) signaling pathway. (**A**) Protein expression levels of ERK1/2 mitogen-activated protein kinases in BV2 cells in response to CXCL3 treatment (10 ng/mL) (western blot). p-ERK1/2, phosphorylated ERK1/2. (**B**) Inducible nitric oxide synthase (iNOS) and cluster of differentiation (CD) 16 protein expression in BV2 cells after 2 h of pre-treatment with U0126 (5 μM) and 12 h of treatment with CXCL3 (10 ng/mL) (western blot). (**C**) mRNA expression of pro-inflammatory markers (tumor necrosis factor-alpha [TNF-α], interleukin [IL]-1β, IL-6, NOS2, and cluster of differentiation 86 [CD86]) in BV2 cells were pretreated with U0126 (5 μM) for 2 h then treated with CXCL3 (10 ng/mL) for 6 h (n = 3; quantitative real-time-polymerase chain reaction [PCR]). GAPDH was used as an internal control for normalization. (**D**) mRNA expression of TNF-α, IL-1β, IL-6, NOS2, and CD86 in the brains of mice were pretreated with U0126 (10 mg/kg; intraperitoneal injection) for 12 h, then infected with meningitic *E. coli* for 5 h (n = 5 per group; real-time PCR). β-actin was used as an internal control for normalization. Data were presented as mean ± SD from five independent assays. *p* < 0.05 (*) was considered statistically significant; *p* < 0.01 (**) and *p* < 0.001 (***) indicated extremely significant differences; ns, not significant.

Recent studies suggest that CXCL3 might activate multiple signaling pathways. Therefore, the intracellular signaling pathways activated by CXCL3 were investigated. Recombinant mouse CXCL3-treated BV2 cells were used to detect intracellular signaling. A rapid phosphorylation of ERK1/2 was induced by CXCL3 (Figure 4A). 

Next, we investigated whether the ERK1/2 pathway was involved in pro-inflammatory factor production. Following pre-treatment with U0126 (a specific ERK1/2 inhibitor), iNOS, and CD16 expression significantly decreased (Figure 4B). In addition, IL-1β, IL-6, TNF-α, and iNOS, as well as CD86 expression substantially decreased to different extents than those in their respective control groups (Figure 4C).

Finally, we performed in vivo experiments, administering U0126 intraperitoneally (10 mg/kg) for 12 h to block ERK signaling before the intravenous injection of meningitic *E. coli.* The subsequent real-time PCR analysis indicated that U0126 treatment decreased IL-1β, IL-6, iNOS, and CD86 expression (Figure 4D). According to these results, ERK1/2 is active and participates in the CXCL3-induced inflammatory responses in BV2 cells. 

### 2.5. CXCR2 Blockade Inhibits the Expression of Pro-Inflammatory Factors

**Figure 5:**
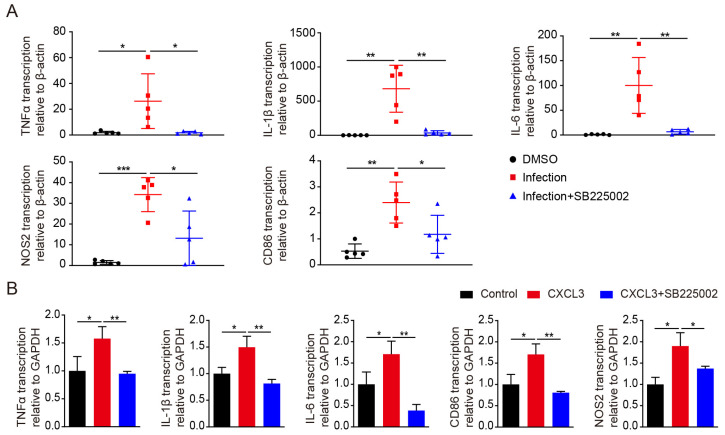
The effects of the C-X-C motif chemokine receptor 2 (CXCR2) antagonist on pro-inflammatory cytokines in vivo and in vitro. (**A**) mRNA expression of tumor necrosis factor-alpha (TNF-α), interleukin (IL)-1β, IL-6, nitric oxide synthase 2 (NOS2), and cluster of differentiation (CD) 86 in the brains of mice that were pretreated with the CXCR2 antagonist SB225002 (1 mg/kg; intraperitoneal injection) for 12 h then infected with meningitic *E. coli* for 5 h (n = 5 per group; was determined by real-time polymerase chain reaction [PCR]). (**B**) mRNA expression of pro-inflammatory markers in BV2 cells were pretreated with SB225002 (50 nM) for 2 h then treated with C-X-C motif chemokine 3 (CXCL3) (10 ng/mL) for 6 h (n = 3; quantitative real-time PCR). β-actin was used as an internal control for normalization. Data were presented as mean ± SD from five independent assays. *p* < 0.05 (*) was considered statistically significant; *p* < 0.01 (**) and *p* < 0.001 (***) indicated extremely significant differences.

CXC chemokines exert their biological roles through the chemokine receptors CXCR1 and CXCR2. To confirm the critical role of CXCL3 in microglia activation, we intraperitoneally injected 1 mg/kg of the CXCR2 antagonist, SB225002 (a specific CXCR2 antagonist), to inhibit CXCL3 activity 12 h before the intravenous injection of meningitic *E. coli.* SB225002 treatment decreased the IL-1β, IL-6, TNF-α, iNOS, and CD86 expression levels (real-time PCR; Figure 5A). We also pre-treated BV2 cells with SB225002, finding similar results to the in vitro experiment (Figure 5B). 

**Figure 6:**
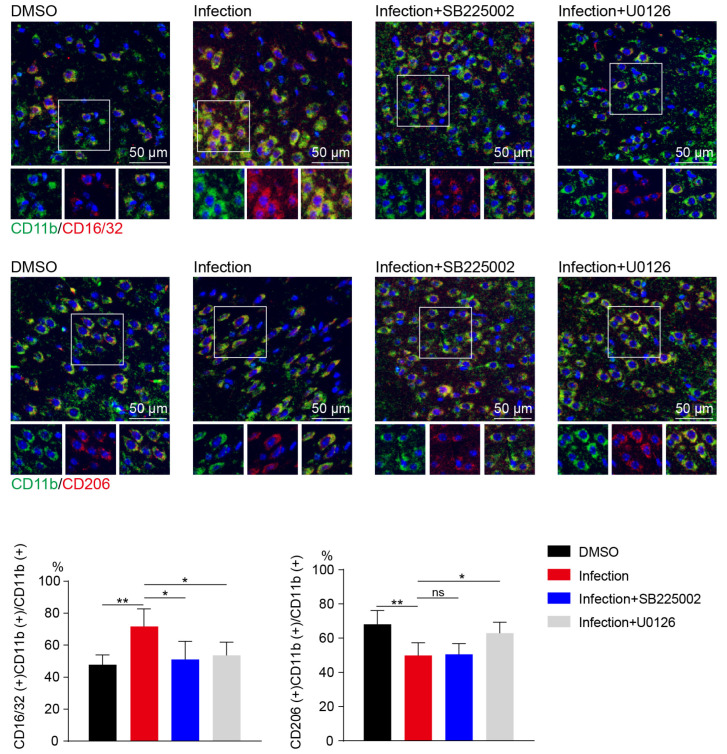
The effect of C-X-C motif chemokine receptor 2 (CXCR2) or extracellular signal-regulated protein kinases 1 and 2 (ERK1/2) antagonists on microglia polarization in vivo. Immunofluorescence results for CD11b (green), CD16/32 (red), and CD206 (red) in the brains of mice were pretreated with SB225002 (1 mg/kg; intraperitoneal injection) or U0126 (10 mg/kg; intraperitoneal injection) for 12 h then infected with meningitic *E. coli* for 5 h. Scale bar, 50 µm. Quantitative analysis of CD11b and CD16/32 double-positive and CD11b and CD206 double-positive cells. Data were presented as mean ± SD from three independent assays. *p* < 0.05 (*) was considered statistically significant; *p* < 0.01 (**) indicated extremely significant differences; ns, not significant.

Finally, immunofluorescence indicated that SB225002 or U0126 treatment significantly decreased the percentage of CD16/32 and CD11b double-positive microglia after meningitic *E. coli* infection compared to those without SB225002 or U0126 treatment (Figure 6). These results further indicated that CXCL3 plays a critical role in *M1* microglia activation.

## 3. Discussion

**Figure 7:**
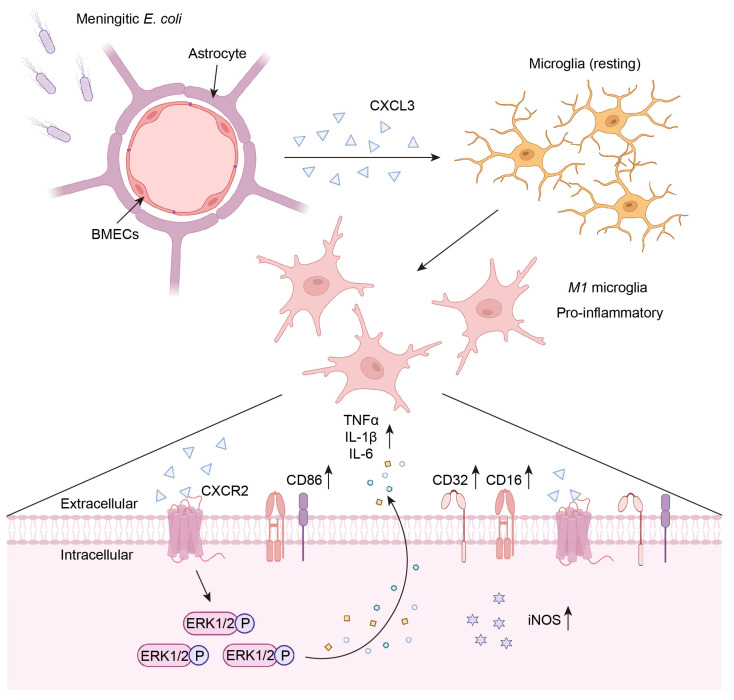
The pathway and molecular schematic diagram of astrocyte and BMEC cell-derived CXCL3 promoting *M1* type differentiation of microglia. Meningitic *E. coli* infection of BMECs and astrocytes induced the upregulation of CXCL3. CXCL3 binds to CXCR2 then promotes microglia *M1* polarization. At the same time, CXCL3 induced ERK1/2 phosphorylation and promoted pro-inflammatory factors expression, resulting in exacerbation of neuroinflammation.

*E. coli* is an important Gram-negative bacterium and an important contributor to meningitis [5,17]. *E. coli* induces inflammation in the CNS by breaking through the BBB. However, the mechanism by which it breaks through the BBB remains unclear. Previous studies have mainly reported the important role of *E. coli* virulence factors in bacterial BBB penetration. In contrast, few studies have examined how host-intrinsic factors may influence the development of meningitis. Our previous study found that infection with meningitic *E. coli* can significantly increase the expression of chemokines and cytokines, such as IL-6, IL-1β, TNF-α, CXCL3, CXCL2, and C-C motif chemokine ligand (CCL) 2, and some chemokines remain at high levels in the blood and brain for a long time [17,19]. In this study, we demonstrated that CXCL3 promotes microglia *M1* polarization by binding to CXCR2, which ultimately induces the expression of pro-inflammatory cytokines (Figure 7). 

CXCL3, an individual that belongs to the CXC chemokine subfamily, is widely expressed and associated with organ injury and inflammatory responses [20]. CXCL3 is highly upregulated during esophageal carcinogenesis and contributes to vascular invasion in gastric cancer and human melanoma. As well as promoting tumor cell migration, CXCL3 is associated with the migration of airway smooth muscle cells and neurons. Furthermore, in preeclampsia, activation of CXCL3 by endogenous factors promotes trophoblast invasion, migration, and proliferation in human trophoblasts and is critical to the pathogenesis of preeclampsia [21]. CXCL3 appears to play a role in several pathophysiological processes. However, the role of CXCL3 in CNS inflammation remains to be investigated. Compared to BMECs, astrocytes release significantly higher levels of CXCL3 in response to stimulation with the meningitis *E. coli* infection, suggesting that astrocytes are the main source of CXCL3, which acts on other cells and causes a series of biological effects.

The biological functions of the chemokines in the CXC family depend on binding to their receptors CXCR1 or CXCR2. CXCR2 is expressed on endothelial cells, oligodendrocytes, and various immune cells; multiple sclerosis, traumatic brain injury, and Alzheimer’s disease are associated with CXCR2 and its ligands [22,23,24]. CXCR2 antagonists have been proposed as a therapeutic strategy for treating inflammatory diseases [25]. Microglia do not express CXCR2 under homeostatic conditions; however, CXCL3 robustly induces high levels of CXCR2 mRNA and protein expression in BV2 microglia, and its expression is upregulated when activated during CNS pathologies. In contrast, in this study, we identified CXCR2 expression in the control group, perhaps because of differences between the cells in vitro and primary cells; the culture environment in vitro also differs from that in vivo. Therefore, we speculate that CXCL3 could either promote or inhibit the inflammatory response in microglia.

Microglia in an *M1* cellular state are pro-inflammatory and associated with an overexpression of inflammatory cytokines, including IL-1β, TNF-α, and iNOS. In addition, they present antigens to T cells by expressing CD80, CD16/32, and major histocompatibility complex class II molecules on their membranes [26]. Conversely, microglia in the *M2* polarized state release beneficial mediators, including IL-4, IL-10, and transforming growth factor-β, and Ym-1 and CD206 are abundant antigen-presenting molecules on their surface, leading to homeostasis, regeneration, and neuroprotection [27]. In response to cerebral ischemia, sphingosine 1-phosphate receptor (S1PR2) activates microglia and induces *M1* polarization through the ERK1/2 and c-Jun N-terminal kinase (JNK) pathways [28,29]. Several studies have shown that the CCL19, CCL21, CCL24, CCL25, CXCL8, CXCL10, and X-C motif chemokine ligand 2 (XCL2) assist *M1* macrophages in chemotaxis, whereas CCL7 accompanies both *M1* and *M2* macrophages in their chemotaxis [30,31,32]. Similarly, this study found that CXCL3 was also involved in *M1* macrophage chemotaxis.

Some studies have shown that CXCL3 promotes prostate cancer cell proliferation and migration by upregulating p-ERK1/2, p-Akt, and Bcl2 and downregulating Bax [33]. Simultaneously, CXCL3 promotes the formation of fat cells by activating the ERK/MAPK and JNK/MAPK pathways [11], which suggests that CXCL3 induces *M1* microglia by selectively activating ERK1/2 MAPK signaling, promoting an inflammatory response.

## 4. Materials and Methods

### 4.1. Bacterial Strains and Cell Culture

In this study, we used a meningitic *E. coli* strain (PCN033) maintained in our laboratory. Luria-Bertani medium was used to grow the bacterial cells.

hBMECs were cultured in RPMI-1640 medium supplemented with 10% fetal bovine serum (FBS), L-glutamine, sodium pyruvate, non-essential amino acids, vitamins, penicillin, and streptomycin (100 U/mL) at 37 °C under 5% CO_2_ [34]. Serum-free medium was used to starve confluent cells for 16–18 h. Dulbecco’s modified Eagle’s medium supplemented with FBS was used to culture the human astrocyte cell line U251 and the mouse microglial cell line BV2 [35].

### 4.2. Meningitic E. coli Infection of hBMECs and U251 Cells

*E. coli* was cultured overnight and diluted in serum-free medium before being added separately to the confluent hBMECs and U251 cell monolayer cultures. Samples were then harvested and processed using TRIzol reagent or cell lysis buffer after three washes with pre-chilled phosphate-buffered saline (PBS).

### 4.3. Western Blotting

After dilution of brain homogenates or cell lysates in loading buffer, they were boiled at 100 °C for 10 min. An acrylamide-sodium dodecyl sulfate gel containing 12% acrylamide was used for loading. Polyvinylidene difluoride membranes were used to transfer the gels. TBST (10 mM Tris-buffered saline with 0.05% Tween 20) was used to block the membranes for 2 h, followed by overnight incubation with either antibody. After washing, a species-specific horseradish peroxidase-conjugated antibody was added, and the blots were visualized with ECL reagent after incubation.

The CXCR2 (rabbit) antibody (20634-1-AP, 1:2000 dilution), β-actin (mouse) antibody (66009-1-Ig, 1:5000 dilution), iNOS (rabbit) antibody (80517-1-RR, 1:1000 dilution), and CD16 (rabbit) antibody (16559-1-AP, 1:2000 dilution) were obtained from Proteintech (Chicago, IL, USA); the ERK (rabbit) antibody (A0229, 1:2000 dilution) was obtained from ABClonal (Wuhan, China); the phospho-ERK1/2 (rabbit) antibody (4370, 1:2000 dilution) was obtained from Cell Signaling Technology (Danvers, MA, USA); the CXCL3 (rabbit) antibody (sc-365870, 1:1000 dilution) was obtained from Santa Cruz Biotechnology (Dallas, CA, USA).

### 4.4. Isolation and Quantitative Real-Time Polymerase Chain Reaction (qRT-PCR) Analysis of RNA

Total RNA was isolated from brain lysates or cells using Trizol reagent (Aidlab Biotech, Beijing, China). HiScript II Q RT SuperMix for qPCR gDNA wiper (Vazyme, Nanjing, China) was used to synthesize complementary DNA from RNA aliquots from each sample. Using MonAmp SYBR Green qPCR Mix (RN04005M, Monad Biotech Co., Ltd. Wuhan, China), real-time PCR was performed on a qTOWER3/G quantitative real-time PCR thermal cycler (Analytikjena, Jena, Germany). The target gene expression was normalized using glyceraldehyde-3-phosphate dehydrogenase (GAPDH) or β-actin as a control.

### 4.5. Secretory CXCL3 Determination by Enzyme-Linked Immunosorbent Assay (ELISA)

Mice were challenged with meningitic *E. coli* as described above. Mice were euthanized at the indicated time points, and the serum was collected and stored at −80 °C. ELISA kits (Elabscience, Houston, TX, USA) were used to measure serum CXCL3 concentrations according to the manufacturer’s instructions.

### 4.6. Flow Cytometry Analysis

Flow cytometry was applied to compare the expression of CD16/32 and CD206 in BV2 cells stimulated by CXCL3. Cells were seeded at a density of 1 × 10^6^ in 6-well plates and cultured for 24 h. The cells were incubated with CXCL3 for 3 h, treated with trypsin without EDTA, and washed with cell staining buffer. Cells were incubated with FITC-conjugated monoclonal rat CD16/32 antibodies at 4 °C for 30 min, washed with a cell staining buffer, and then incubated with a fixation buffer at room temperature for 20 min. Next, the cells were treated with a permeabilization wash buffer then incubated with APC-conjugated monoclonal rat CD206 antibodies at 4 °C for 30 min and washed with a cell staining buffer. Finally, the cells were washed with a permeabilization wash buffer and suspended in a cell staining buffer in the dark at 4 °C. CD16/32 and CD206 expression levels were analyzed using a FACS Calibur Flow cytometer (Becton Dickinson, Franklin Lakes, NJ, USA). The FITC anti-CD16/32 (FITC-65080) and APC anti-CD206 (APC-65155) were obtained from Proteintech (Chicago, IL, USA).

### 4.7. Immunofluorescence Analysis

Mice with the typical CNS disorder were given ketamine-xylazine (0.1 mL/10 g) and perfused with PBS. Brain samples were then removed, fixed in 4% formaldehyde solution, and embedded in paraffin.

Sections were incubated with the primary antibody, conjugated with either Cy3 or FITC, and finally incubated with the appropriate secondary antibody, DAPI, to stain the nucleus.

The following antibodies were used: anti-CD31 (ab28364, 1:200 dilution), anti-CD11b (ab184308, 1:100 dilution), anti-CD206 (ab64693, 1:100 dilution), and anti-CD16 (ab246222, 1:100 dilution) were obtained from Abcam (Cambridge, MA, USA); FITC-labeled goat anti-mouse antibody (A0568, 1:200 dilution), Cy3-labeled goat anti-rabbit antibody (A0521, 1:200 dilution), and DAPI (C1005) were obtained from the Beyotime Institute of Biotechnology (Shanghai, China).

### 4.8. Animal Infection Assay

Four-week-old Kunming (female) mice were obtained from the Laboratory Animal Center of China at Three Gorges University (Wuhan, China) for animal infection studies. These mice were then injected with meningitic *E. coli* through the tail vein with 1 × 10^7^ colony-forming units (CFUs) and then sacrificed. At the designated times, the mice were anesthetized, their peripheral blood was collected for serum extraction, and it was then processed for further testing.

SB225002 (HY-16711, MedChemExpress; Summit, NJ, USA) is a selective non-peptide CXCR2 (the CXCL3 receptor) inhibitor and was used to inhibit CXCL3 activity. To block CXCR2, SB225002 (1 mg/kg) was injected intraperitoneally into mice 12 h before the intravenous injection of meningitic *E. coli*; a similar amount of DMSO was injected into the control group.

### 4.9. Statistical Analyses

Data were expressed as means ± standard deviations unless otherwise specified. Between-group differences were analyzed using one-way analysis of variance (ANOVA) in GraphPad Prism version 7.0 (GraphPad Software, La Jolla, CA, USA). *p*-values of < 0.05 indicated significant differences, and those <0.01 and <0.001 indicated extremely significant differences.

## 5. Conclusions

In conclusion, our results indicate that meningitic *E. coli* infection promotes CXCL3 expression, and CXCL3 binds to CXCR2 to promote neuroinflammation by regulating downstream signaling pathways and expression of pro-inflammatory factors in microglia. These findings suggest that CXCL3 may be a novel therapeutic target for *E. coli*-induced CNS inflammation.

## Data Availability

Not applicable.
